# Characteristics of Serotype 3 Invasive Pneumococcal Disease before and after Universal Childhood Immunization with PCV13 in Massachusetts

**DOI:** 10.3390/pathogens9050396

**Published:** 2020-05-21

**Authors:** Rotem Lapidot, Kimberly M. Shea, Inci Yildirim, Howard J. Cabral, Stephen I. Pelton, the Massachusetts Department of Public Health

**Affiliations:** 1 Division of Pediatric Infectious Diseases, Boston Medical Center, Boston, MA 02118, USA; spelton@bu.edu; 2 Boston University Schools of Medicine, Boston, MA 02118, USA; 3 Boston University School of Public Health, Boston Medical Center, Boston, MA 02118, USA; Kimberly.Shea@pfizer.com; 4 Division of Pediatric Infectious Diseases, Department of Pediatrics, Emory University School of Medicine, Atlanta, GA 30322, USA; inci.yildirim@emory.edu; 5 Department of Epidemiology, Rollins School of Public Health, Emory University, Atlanta, GA 30322, USA; 6 Department of Biostatistics, Boston University School of Public Health, Boston, MA 02118, USA; hjcab@bu.edu; 7 Massachusetts Department of Public Health, 250 Washington St, Boston, MA 02108, USA

**Keywords:** *Streptococcus pneumoniae serotype 3*, PCV13, invasive pneumococcal disease

## Abstract

Background: Although a substantial decline in vaccine-serotype invasive pneumococcal disease (IPD) incidence was observed following the introduction of pneumococcal conjugate vaccines (PCV), the estimated range of thirteen-valent conjugate vaccine (PCV13) effectiveness for serotype 3 disease is wide and includes zero. We assessed the impact of PCV13 on serotype 3 IPD incidence and disease characteristics in Massachusetts’ children. Methods: Serotype 3 IPD cases in children <18 years old were identified via enhanced passive surveillance system in Massachusetts. We compared incidence rates and characteristics of IPD cases before and after PCV13. Results: A total of 47 serotype 3 IPD cases were identified from 2002 to 2017; incidence of serotype 3 IPD in the years following PCV13 was 0.19 per 100,000 children compared to 0.21 before PCV 13, incidence rate ratio (IRR) = 0.86 (95% CI 0.47–1.57). The majority (78%) of post-PCV13 serotype 3 IPD cases occurred among fully vaccinated children. Age distribution, clinical syndrome and presence of comorbidities among serotype 3 IPD cases were similar before and after PCV13 introduction. There was no association between the date of the last PCV13 dose and time to IPD to suggest waning of immunity. Conclusions: seven years following PCV 13 we found no significant changes in serotype 3 IPD incidence or disease characteristics in children in Massachusetts.

## 1. Introduction

The introduction of pneumococcal conjugate vaccines has dramatically decreased the incidence of invasive pneumococcal disease (IPD) in children [1]. In the United States, the introduction of the seven-valent pneumococcal conjugate vaccine (PCV7) in 2000 resulted in a substantial reduction of vaccine-type and overall IPD incidence [2]. However, soon after the introduction of PCV7, an increase in IPD due to non-vaccine serotypes was observed, prompting the development of expanded serotype conjugate vaccines. The thirteen-valent conjugate vaccine (PCV13) contains six additional serotypes beyond those in PCV7, one of which is serotype 3. PCV13 was introduced in Massachusetts in April 2010 and resulted in further decline in overall IPD incidence in children [3].

Pneumococcus serotype 3 has many unique biological properties compared to other pathogenic pneumococcal serotypes; it has a thick capsule surrounded by a slime layer which gives it a characteristic mucoid appearance on blood agar plates and enables it to resist in vitro opsonophagocytosis. It has a high invasive capacity [4,5] and causes severe infections such as complicated pneumonias and empyema, with high mortality rates [6]. Serotype 3 is a common cause of invasive diseases in the adult population, accounting for as much as 15% of IPD cases in some reports [7].

The immunologic response induced by the conjugate vaccine to serotype 3 polysaccharide appears to differ from the response induced by other vaccine serotypes. Clinical trials of PCV13 immunogenicity demonstrated that the humoral immune response to serotype 3 was lower compared to the other five new serotypes [8,9]. Whether lower immunogenicity translates into less effectiveness against serotype 3 has yet to be determined.

In the pre-PCV era, serotype 3 IPD in children occurred with relatively low frequency, and incidences varied greatly from year to year and without an apparent predictive pattern. Serotype 3 colonization rates in the pediatric population were similarly relatively low [10]. Because serotype 3 occurs infrequently, it has been challenging to assess the post licensure clinical impact of PCV13 on the incidence of serotype 3 IPD. Several years after PCV7 was replaced by PCV13 in routine infant immunization programs across the world, there was growing evidence of a variable impact on serotype 3 IPD in different settings or potentially at different times [9,11]. In this analysis, we utilized data from an existing long-term pneumococcal surveillance program in Massachusetts’ children to describe the impact of PCV13 on serotype 3 IPD incidence and disease characteristics.

## 2. Results

**Figure 1:**
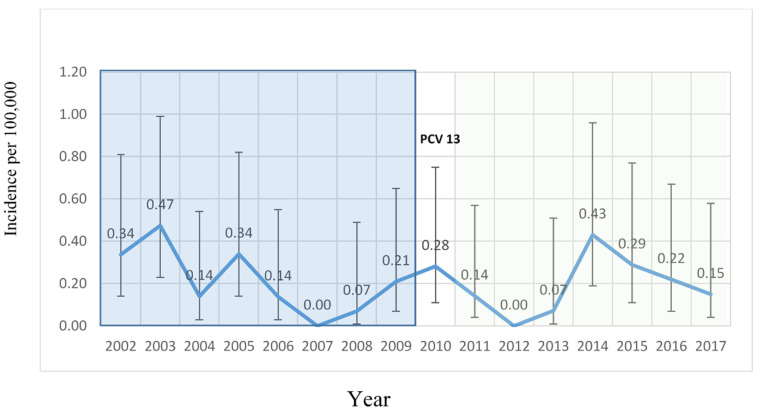
Annual incidence of serotype 3 invasive pneumococcal disease (IPD) cases in Massachusetts’ children.

A total of 1171 cases of IPD in children younger than 18 years old residing in Massachusetts were reported between 2002 and 2017. Of these, 47 (4%) were due to serotype 3 (Figure 1). 

Selected demographics of serotype 3 IPD cases are shown in Table 1. 

Twenty-five cases of serotype 3 IPD occurred over the eight-year period prior to the introduction of PCV13 (January 2002 to December 2009). Eighteen cases occurred over the seven-year period following the introduction of PCV13 (January 2011 to December 2017). Four serotype 3 cases occurred during 2010, the year PCV 13 was introduced, and were excluded from this analysis. Among the post- PCV13 cases, 14 cases (78%) occurred in children who were fully vaccinated for their age, two (11%) occurred in infants aged 0–2 months who had not yet received their routine two-month vaccine, and two (11%) cases were in older unvaccinated children. 

The incidence of serotype 3 IPD per 100,000 children did not significantly change over time. The mean incidence rate after PCV13 and before PCV13 was 0.19 and 0.21, respectively, with an incidence rate ratio [IRR] of 0.86 (95% CI 0.41–1.82). The yearly trend of serotype 3 IPD was decreasing [−0.04 (95% CI −0.09, 0.0051)] prior to PCV 13 and increasing [0.07 (95% CI −0.0087, 0.14)] in the years following PCV13. The change in trend of serotype 3 IPD incidence before and after PCV13 was not significant [post-PCV13 vs. Pre-PCV13 was 0.00017 (95% CI −0.37, 0.37)]. The characteristics of children who had serotype 3 IPD were generally comparable across the two time periods. The age distribution at the time of IPD was similar before and after PCV13, with the majority of cases occurring in the young population (Table 1). There was also a similar distribution of IPD clinical syndromes before and after PCV13 (56% and 55.6% of cases were bacteremic pneumonia/empyema, 28% and 27.7% isolated bacteremia, 12% and 11.1% meningitis before and after the introduction of PCV13, respectively). The time from the last PCV7 dose to serotype 3 IPD ranged from 9 to 1521 days (median 222 days), and from last PCV13 dose to serotype 3 IPD date ranged from 23 to 1243 days (median 432 days) and was not statistically different between the two study periods (Wilcoxon two–sample test *p* = 0.26).

Within the population of children that had serotype 3 IPD in the post-PCV13 era, three (16.7%) had an underlying comorbidity compared to two (8%) children with underlying comorbidities in the pre-PCV13 era (these comorbidities include cerebral palsy, chronic lung disease, congenital heart disease, prematurity/low birth weight and sickle cell disease), relative risk ratio [RR] = 2.08 (CI 95% 0.39–11.22).

## 3. Discussion

Our analysis of IPD in Massachusetts’ children between 2002 and 2017 did not identify any significant decrease or increase in serotype 3 IPD incidence rates or change in disease characteristics after PCV13 replaced PCV7, though most children who developed serotype 3 IPD in the post-PCV13 period were fully immunized. We observed a similar age distribution of IPD occurrence before and after the introduction of PCV13, comparable clinical syndromes, and comparable time to disease after vaccination either with PCV7 (which does not contain serotype 3 capsular antigen) or PCV13 (which contains serotype 3 capsular antigen). We did observe an increase in the proportion of children with underlying comorbidities in the post-PCV13 era, which is consistent with what we reported for overall IPD in Massachusetts’ children [12]. Our findings support the hypothesis that there was no decline in serotype 3 IPD incidence or change in disease characteristics in Massachusetts’ children following PCV13 introduction. Other studies have reported similar observations; in Denmark no change beyond what would be expected from natural cyclic patterns was observed in serotype 3 IPD three years following the introduction of the PCV13 vaccine [13], as was the case in Israel [14]. In Italy a small decrease was observed [15]. In a systematic review of PCV13 vaccine effectiveness for serotype 3, an overall reduction in population based incidence was reported early after the vaccine was introduced, but not sustained [16]. In England and Wales serotype 3 IPD incidence fluctuated over a period of 17 years, with an increase in incidence seen three years following the introduction of PCV13 [17]. In Spain, there were no significant changes in incidence of serotype 3 IPD following the vaccine [18], in Germany and Hong Kong an increase in serotype 3 empyema and IPD, respectively, was reported [19,20].

There are several possible hypotheses that could explain why PCV13 fails to impact serotype 3 invasive disease incidence. Inadequate immunogenicity of the serotype 3 capsular antigen, possibly as a result of intrinsic resistance to opsonic killing [21,22,23], waning of immunity to serotype 3 over time, a lack of protection against colonization with serotype 3 pneumococcus, resulting from failure of PCV13 to induce mucosal immunity are all potential explanations for the observed lack of reduction.

In pre-licensure studies of PCV13, detectable antibodies to serotype 3 using an Enzyme Immunosorbent Assay (ELISA) were present following immunization, but post-immunization geometric mean concentrations were lower compared to other vaccine serotypes, and did not meet the prespecified criteria for seroresponsiveness. However, opsonophagocytosis assays (OPA) demonstrated 98% of subjects had titers of 1:8 or higher [8]. The immune response to serotype 3, as measured by OPA, was lower compared to the majority of vaccine serotypes following immunization [24]. Furthermore, the immunologic response of serotype 3 was not augmented consistently by booster doses, unlike other serotypes included in the conjugate vaccines [8,25]. Post-licensure studies of the effectiveness of PCV13 demonstrated low effectiveness against serotype 3 invasive diseases [9,26,27,28]. Andrews et al. reported that IgG levels that correlated with protection against serotype 3 IPD are substantially higher than the currently accepted 0.35 µg/mL correlation of protection [9]. Such high levels of antibodies may be difficult to achieve, and even more challenging to sustain following immunization. This could explain why an 11-valent vaccine also failed to protect against acute otitis media caused by serotype 3 (as opposed to all other 10 serotypes tested) [29]. If the vaccine induced brief, transient protection we would have expected a decrease in serotype 3 IPD cases occurring in late infancy, and a shift of the disease towards older patients. We did not observe this in our population, nor did we observe an effect on time from the last vaccine dose to the development of serotype 3 IPD between children immunized with PCV13 compared to those with PCV7. These observations fail to support a hypothesis of initial protection followed by waning immunity as would be manifested by an increase in age at the time of IPD or delayed onset of serotype 3 cases after immunization with PCV13.

To estimate the true effect of the vaccine on the burden of pneumococcal diseases, non-invasive diseases should also be taken into account. Acute otitis media and non-bacteremic pneumonia are the most common presentations of pneumococcal disease in the pediatric population. In adults for example, it is estimated that non-bacteremic pneumococcal pneumonia incidence is more than three times higher than that of bacteremic pneumonia [30,31].

Our surveillance data included only invasive pneumonia (i.e., bacteremic pneumonia and pneumonia with empyema). Is it possible that we are overlooking an effect of the vaccine on non-invasive pneumonia in our population? Biologically, the immune response needed to protect against non-invasive disease syndromes (pneumonia and otitis media) is thought to be higher than that needed for protection against invasive diseases. Therefore, if PCV13 had induced protective concentrations of antibodies against non-bacteremic pneumonia, we would expect these levels to be high enough to protect against invasive diseases, and we should have been able to detect an effect on IPD incidence, which we did not. In addition, if the immunologic response induced by PCV13 against serotype 3 was sufficient to protect against bacteremia but not pneumonia, which is hypothesized to require higher concentrations of antibodies, we would have expected to see a proportional decline in bacteremia compared to pneumonia rates, which did not occur.

The lack of effect of the vaccine on nasopharyngeal colonization might be another explanation from our observations. A three-fold increase in serotype 3 nasopharyngeal colonization prevalence was reported in children younger than seven years of age in the initial years following vaccine’s introduction [32]. In a double-blind pre-licensure clinical trial, serotype 3 nasopharyngeal acquisition did not differ between children immunized with PCV7 and those immunized with PCV13 [33]. Cohen et al. [34] observed a decreasing trend in carriage of serotype 3 over 13 years of follow up in children younger than 24 months with acute otitis media, but it was not correlated with the implementation of PCV13. It might be that protection against colonization is achieved by immunological pathways (such as the activation of Th-17) that are not induced by this serotype 3 polysaccharide conjugate formulation, or that higher antibody responses are needed for mucosal protection than those achieved by the vaccine.

One of the most striking and important characteristics of PCV7 was its effect on the reduction of IPD in the non-targeted population, e.g., elderly population, achieved by immunizing the children [35]. The effect of PCV13 on overall IPD cases was also attributed mainly to indirect protection in the unvaccinated population, as with the case of PCV7 [36]. However, in Denmark, four years following routine childhood vaccinations with PCV13, the incidence of serotype 3 IPD cases in the elderly population was not lower [37]. The same observation was noted in Norway, where even though early after introduction of the vaccine no serotype 3 IPD cases were diagnosed in children younger than 5 years of age, there was no change in serotype 3 IPD incidence in the non-targeted age groups [38]. In Canada, an increase in hospitalized adults with community acquired pneumonia due to pneumococcus serotype 3 masked the impact of herd immunity the vaccine achieved for other strains covered by the vaccine [39]. The impact of the vaccine on the non-targeted population is particularly significant in the case of serotype 3, which has a high incidence of serious diseases in the adult population. The lack of indirect protection might be because the vaccine is not inducing high enough levels of antibodies, but also could be because we are not targeting the correct population; there are low colonization rates of serotype 3 in the pediatric population, and it may be that serotype 3 is carried disproportionately in the adult population. Interestingly, colonization rates with serotype 3 in Massachusetts’ children increased following the introduction of PCV13, while overall colonization rates with *S. pneumoniae* remained stable around 30% for children younger than seven years [32]. Although the most likely explanation is cyclic, if this increase in colonization persists, one explanation could be hyporesponsiveness to the vaccine, where repeated exposures to the vaccine create tolerance, and a reduced antibody response [40,41].

Our study describes important clinical and demographic characteristics of serotype 3 IPD cases, adding further evidence to the reported lack of impact of PCV13 on serotype 3 IPD using a long-standing population based statewide surveillance, not limited to hospitalized patients.

Our study has several limitations that should be considered. As expected with serotype 3, we observed a small number of IPD cases over our nearly 15-year study. Therefore, our study may be underpowered to detect differences between the two time periods compared in this study. In addition, given the high variability of serotype 3 IPD incidence from year to year, it is possible that we need a longer observation period. If a small effect of PCV13 on nasopharyngeal serotype 3 carriage does exist, it may take up to ten years to see a reduction of IPD incidence [42], suggesting that six years of post-PCV13 observation may not be sufficient to detect changes in serotype 3 IPD. However, we believe that our observation is consistent with other studies demonstrating relatively low effectiveness and impact of the vaccine against serotype 3 IPD in the pediatric population [13,14,15].

Another limitation of our study is a lack of data regarding IPD incidence in adults as adult IPD is not a reportable disease in Massachusetts. Further studies are needed to continue to assess the impact of PCV13 on serotype 3 invasive disease and colonization, both in the pediatric population as well as the adult population. Studies of the immunological pathways induced by serotype 3 disease and colonization may shed more light on the reasons for the lack of impact of the vaccine.

## 4. Materials and Methods

Study design and population:

Study population: We identified serotype 3 IPD cases from an ongoing population-based enhanced passive surveillance system for IPD occurring in children younger than 18 years of age in Massachusetts from January 2002 through to December 2017. All pediatric cases of IPD, defined as a child with a positive culture for *Streptococcus pneumoniae* from a sterile body site, are reportable to the Massachusetts Department of Public Health (MDPH). Health Department epidemiologists interview caregivers and/or primary care providers of all reported cases to obtain demographic and clinical information via a standardized case report form for every reported case of IPD. All available isolates of *S. pneumoniae* from a sterile body site (IPD cases) were sent by MDPH to the Maxwell Finland Laboratory for Infectious Diseases at Boston Medical Center and were serotyped using the Quellung reaction with pneumococcal antisera (Statens Serum Institute, Copenhagen, Denmark).

Statistical analysis: We calculated annual incidence rates of serotype 3 IPD by dividing the number of serotype 3 IPD cases reported to the MDPH each year by the US census estimates of the Massachusetts’ pediatric population, and expressed incidence per 100,000 children. Because PCV13 was introduced in Massachusetts in April 2010, we designated 2010 as a transition year and excluded this year from our analysis. To assess the impact of PCV13 on serotype 3 IPD we compared the incidence of serotype 3 IPD before (2002–2009) and after (2011–2017) the introduction of PCV13 via interrupted time series analysis and a chi-square test. We also compared the age distribution, clinical syndromes, immunization status and prevalence of underlying comorbidity among IPD cases between the two study periods. We compared the time from the last PCV dose to the IPD diagnosis between cases vaccinated only with PCV7 and those vaccinated with PCV13 using the Wilcoxon two–sample test. For the purpose of this analysis, we defined fully immunized children for PCV13 as those who received the recommended number of vaccine doses for their age or if they received a catch up dose of PCV13 as per CDC recommendations [43].

All analyses were performed in MS Excel and SAS software version 9.4 (SAS Institute, Cary, NC, USA).

The Boston University Medical Center IRB reviewed the study and determined it was exempt status.

## 5. Conclusions

We found no impact of PCV13 on serotype 3 IPD incidence or disease characteristics in the pediatric population in Massachusetts. The reported increase in serotype 3 colonization rates in children followed by PCV13 introduction might provide a clue to the possible explanations to the observed lack of impact.

## Figures and Tables

**Table 1:** Baseline characteristics of serotype 3 IPD cases in Massachusetts in the years 2002–2017.

Characteristic	Serotype 3 Cases Prior to PCV 13 (*n* = 25)	Serotype 3 Cases Following PCV 13 (*n* = 18)
**Age (month), median (range)**	26 (2–149)	31 (0–209)
**≤6**	6 (24%)	2 (11.1%)
**6–≤12**	3 (12%)	0 (0.0%)
**12–≤24**	3 (12%)	5 (27.8%)
**24–≤36**	2 (8%)	4 22.2%)
**>36**	11 (44%)	7 (38.9%)
**Gender, Male *n* (%)**	14 (56%)	11 (61%)
**Race/ethnicity *n* (%)**		
**Asian**	0 (0.0%)	1 (5.6%)
**African American**	1 (4.0%)	4 (22.2%)
**Hispanic**	7 (28.0%)	4 (22.2%)
**White**	11 (44.0%)	8 (44.4%)
**Other/Unknown**	6 (24.0%)	1 (5.6%)
**Immunization status with PCV 13 *n* (%)**		
Fully immunized *	0 (0.0%)	14 (77.8%)
**Partially immunized ^+^**	0 (0.0%)	0 (0.0%)
**No vaccination with PCV 13**	25 (100%)	4 (22.2%)
**IPD syndrome *n* (%)**		
**Bacteremia without a focus**	7 (28.0%)	5 (27.7%)
**Bacteremia with focus**	0 (0.0%)	1 (5.6%)
**Bacteremic pneumonia/Empyema**	14 (56.0%)	10 (55.6%)
**Meningitis**	3 (12%)	2 (11.1%)
**Osteoarthritis**	1 (4%)	0 (0.0)
**Mortality *n* (%)**	0(0.0%)	2 (11.1%)
**Comorbidities ^#^*n* (%)**	2 (8.0%)	3 (16.7%)

PCV13—13-valent Pneumococcal conjugated vaccine; IPD—Invasive pneumococcal disease. * Fully immunized is defined as 2–3 doses of vaccine at 2, 4, 6 months of age and one dose after 12 months of age OR at least one dose after 24 months of age (as per CDC recommendations). + Partially immunized is defined as receipt of at least one dose of PCV 13, without being fully immunized. # Comorbidities included cerebral palsy, chronic lung disease, congenital heart disease, prematurity/low birth weight and sickle cell disease.
